# Development of glioblastoma organoids and their applications in personalized therapy

**DOI:** 10.20892/j.issn.2095-3941.2023.0061

**Published:** 2023-06-05

**Authors:** Can Xu, Xiaoye Yuan, Pengyu Hou, Ziru Li, Changsheng Wang, Chuan Fang, Yanli Tan

**Affiliations:** 1 School of Clinical Medicine, Hebei University, Department of Neurosurgery, Affiliated Hospital of Hebei University, Baoding 07100, China; 2 School of Basic Medical Sciences, Hebei University, Baoding 07100, China; 3 Hebei Key Laboratory of Precise Diagnosis and Treatment of Glioma, Baoding 071000, China; 4 Department of Pathology, Affiliated Hospital of Hebei University, Baoding 07100, China

**Keywords:** Glioblastoma, organoids, glioma stem cell, drug screening

## Abstract

Glioblastomas (GBMs) are the brain tumors with the highest malignancy and poorest prognoses. GBM is characterized by high heterogeneity and resistance to drug treatment. Organoids are 3-dimensional cultures that are constructed *in vitro* and comprise cell types highly similar to those in organs or tissues *in vivo*, thus simulating specific structures and physiological functions of organs. Organoids have been technically developed into an advanced *ex vivo* disease model used in basic and preclinical research on tumors. Brain organoids, which simulate the brain microenvironment while preserving tumor heterogeneity, have been used to predict patients’ therapeutic responses to antitumor drugs, thus enabling a breakthrough in glioma research. GBM organoids provide an effective supplementary model that reflects human tumors’ biological characteristics and functions *in vitro* more directly and accurately than traditional experimental models. Therefore, GBM organoids are widely applicable in disease mechanism research, drug development and screening, and glioma precision treatments. This review focuses on the development of various GBM organoid models and their applications in identifying new individualized therapies against drug-resistant GBM.

## Introduction

Gliomas are the most common brain tumors with the poorest prognosis. Notably, fast-growing and aggressively malignant WHO grade 4 glioma, referred to as glioblastoma (GBM), exhibits only a 3%–7% 5-year survival rate, even with standard surgical treatment combined with chemoradiotherapy. Although human disease research performed in clinical patients is the most appropriate and reliable, ethical limitations and complicated research protocols often prevent such studies from being performed. Therefore, reproducible, reliable alternative *in vivo* and *in vitro* research models must urgently be developed to reveal the disease mechanisms underlying GBM, and facilitate drug discovery and screening. The primary traditional disease models include cultured or patient-derived cell lines and animal models. The cell models usually represent 2-dimensional (2-D) features of cytotoxicity and therefore do not include the effects of any disease-associated dysfunctional inter-cellular communication, physicochemical stimulation, and other critical microenvironmental factors that can be studied *in vivo*. Although animal models can simulate the physiological and pathological processes of multi-cell, multi-tissue, and organ interactions, some human disease phenotypes are difficult to mimic in animals, because of differences in species and genetic backgrounds, and may even yield results inconsistent with actual human pathology. Therefore, disease models with similar structural and functional characteristics to human tissues and organs are urgently needed. Organoids are currently the most promising tool to closely mimic human diseases and simultaneously overcome several limitations of 2-D cell culture models^1^.

Human brain organoid technology, which was first described in 2013, recapitulates the characteristics of brain development with a high degree of spatial and temporal fidelity, and accurately reproduces brain microstructure. Brain organoids are becoming increasingly valuable for studying normal cortical neurogenesis and various congenital human brain diseases, and have gradually facilitated the translation of basic science research to clinical applications. For example, glioma organoids have been used to study tumor biology and drug response, and are progressively being applied to investigate other neurosurgery-associated diseases^2^. GBM-induced organoids (GBOs), particularly patient induced pluripotent stem cell (iPSC)-derived glioma organoids, are more practically relevant than animal models for assessing the drug sensitivity of glioma cells. GBOs address the major problem of the absence of human therapeutic targets in rodent disease models, and also provide advantages of short culture times and cost savings^3^. GBOs are an attractive GBM drug testing platform for 3 reasons. First, the rapid generation of GBOs enables testing of drugs before clinical application, thus enabling truly personalized therapy. Second, a summary of GBM heterogeneity in individual GBOs has suggested that GBOs may be more efficient than traditional GBM models for screening drug response. Third, the size and diversity of the GBO biobank, and the relative ease of its further expansion, aid in understanding the relationships between GBO genotype and cellular response status^4^. Brain organoids are likely to retain histologic and genomic features of primary tumor-associated lesions after multiple passages, thus revealing intra- and inter-patient heterogeneity, and can be genetically modified to investigate disease mechanisms in these models. Brain organoids can also be used in drug screening assays and in evaluating the responses of tumor subtypes to different drug treatments, including acquired chemoresistance in recurrent diseases^5^. Organoids maintain tumor heterogeneity and drug-resistant cell types during successive passaging, thereby presenting drug-sensitive or resistant phenotypes and mimicking patient responses. Because they yield consistent and accurate results in drug resistance testing, organoid technologies have found a wide range of applications in drug discovery and screening^6^. This review discusses current methods used to establish GBO models and describes research progress in diagnosis and treatment of drug-resistant gliomas. Finally, current bottlenecks in GBO methods and efforts to overcome these difficulties are discussed. The characteristics of the different GBM models are shown in **Table 1**.

**Table 1:** Summary of characteristics of different GBM models

GBM model	Cell resource	Culture condition	Characteristics	Advantages and disadvantages	References
2-D model	Patient-derived GBM cells	DMEM or DMEM/F12 culture medium with 10% FBS	Monolayer adherent cells	Easy cultivation, lack of stemness and differentiation ability	^7,8^
				Sensitivity to therapy and radiation	^10^
3-D model	Established cell line	DMEM/F12 medium without serum, supplemented with EGF and bFGF	Tumor balls	Stemness, self-renewal, and differentiation ability; slow proliferation	^7–9^
				Enhanced heterogeneity and drug resistance	^11,12^
GBO	iPS cells	DMEM/F12 containing B27, N2, and bFGF	Brain tissue organoid	Characteristics of human brain development	^1,13^
	GBM cell line		Matrix-free	High stemness and strong cell-cell interaction	^15^
Genetically manipulated cerebral organoids	iPS cells	DMEM/F12 containing B27, N2, and bFGF	Applied CRISPR/Cas9 technology	Observation of the earliest steps of tumorigenesis in a human context with a defined genetic manipulation	^17–19^
			Organoids derived from patients with c-Met mutation	Accelerated differentiation into neurons for c-Met iPSC-A compared with control iPSC-A	^20^
PDO	Patient tumor tissue	DMEM/F12 containing B27, N2, and bFGF	Organoids derived directly from glioblastoma specimens	Generation of gradients of stem cell density and hypoxia with PDOs	^22^
		DMEM/F12, Neurobasal medium, 1× GlutaMAX, NEAAs, penicillin–streptomycin, N2, and B27	Preservation of key characteristics and gene expression of the parental tumors	Fast, recapitulation of heterogeneity and key features of glioblastomas	^23–25^
		5% (w/v) GelMA and 0.25% (w/v) HA as the ECM	Application of GelMA–HA hydrogels	Maintenance of parental tumor features, such as the expression of key genes	^26^
		DMEM, FBS, UltraGlutamine I, penicillin–streptomycin, and NEAA	Description of specific steps	High success rate and favorable preservation of patient heterogeneity	^27,28^
			Analysis of genetic characteristics of parental patients and PDO models	Mimicking of the tumor microenvironment and angiogenesis; high-throughput drug screening for precision medicine	^29,30^
GLICO	mESCs and GSCs	mESCs: DMEM-HG, KRS, MEM-NEAA, glutamine, β-mercaptoethanol, and LIF GSCs: Neurobasal medium, B27, N2, bFGF, and EGF	GBM spheroids in coculture with mouse embryonic stem cell (mESC)–derived early-stage cerebral organoids (eCOs)	Favorable simulation of organoid compartments and infiltration patterns	^31^
	GSCs and hESCs	GSCs: Neurobasal medium, B27, N2, bFGF, and FGF Cerebral organoids: DMEM/F12, Neurobasal medium, N2, B27, insulin, GlutaMAX, and MEM-NEAA	GSCs co-cultured with cerebral organoids	Simulation of invasion patterns and chemoradiotherapy resistance of patients with GBM	^10,32^
	iPSCs and GSCs	GSCs: Neurobasal medium, B27, L-Glutamine, Heparin, bFGF, and EGF iPSCs: DMEM/F12 GlutaMAX, N2, B27, insulin, and L-glutamine		Characterization of GBM invasion into human brain at a quantitative or transcriptional level	^33^
3-D printed GBO	GBM cells, ECM, and HUVECs	GBM cells: DMEM, FBS, and penicillin–streptomycin HUVECs: endothelial cell growth medium 2	GBOs reconstructed from patient-derived glioma cells, vascular endothelial cells, and extracellular matrix, on the basis of 3-D printing technology	Recapitulation of the structural, biochemical, and biophysical properties of native tumors; reproduction of clinically observed patient-specific resistance to treatment	^37,38^
	GBM cells, monocytes, GASCs, and microglia	GBM cells and monocytes: MEM, FBS, L-glutamine, and NEAA GASCs: DMEM-HG medium Microglia: microglia complete medium with serum	Use of bioinks based on modified alginate to prepare tumor models incorporating tumor and stromal cells from glioblastoma	Spatial organization of multiple cell types and recovery of protein and RNA at the single cell level	^39^
	GSCs, macrophages, astrocytes, and neural stem cells	GSCs: Neurobasal medium, B27, L-glutamine, bFGF, and FGF Monocytes: RPMI 1640 medium, and FBS Neural stem cells: complete NBM medium for GSCs Astrocytes: astrocyte medium	Model comprising patient-derived GSCs, macrophages, astrocytes, and neural stem cells in HA-rich hydrogel	Recapitulation of glioblastoma transcriptional profiles; promotion of hypoxic and invasive signatures; platform for drug response modeling	^42,43^

## Establishment of GBO models

### 2- and 3-dimensional (3-D) cell models of GBM

In recent decades, cell lines such as U87, U251, and T98 have been widely used as GBM cell models. *In vitro* culture of these cell lines with serum-containing medium can alter their original genomic and transcriptomic profiles, thus resulting in a loss of stemness properties. After implantation into the mouse brain, GBM cells do not recapitulate the classical tumor phenotypes of diffuse infiltration, micro-angiogenesis, and necrosis extending to the surrounding healthy tissues; instead, they form capsular tumors in the surrounding tissues^7,8^. Although extensive research has been conducted in 2-D cell models, these models are increasingly being recognized not to reproduce the multifaceted characteristics of GBM. Consequently, many studies have used patient-derived cell lines maintained under serum-free conditions, nerve cell-specific medium, and low passage conditions to preserve their original phenotypes and genotypes^9^. Linkous et al.^10^ have found that glioma cells grown in 2-D culture are sensitive to alkylating agents and ionizing radiation, but the brain tumors developed through *in vivo* grafting of these cells show substantial therapeutic resistance. Another study has indicated that temozolomide (TMZ) resistance in GBM 3-D cultures is 50% higher than that of a 2-D GBM model, thus highlighting the importance of the extracellular matrix (ECM) in regulating the properties of GBM cells^11^. In addition, the effects of 3 tumor growth inhibitors in established 2-D glioma cells as well as a 3-D *ex vivo* organoid model have indicated that the efficacy of inhibitors is highly correlated with intra- and intertumoral heterogeneity in GBM^12^. To address the shortcomings of previous *in vitro* and *in vivo* GBM models, researchers have focused on developing organoid culture systems that closely mimic the characteristics of GBM, thus providing an opportunity for a powerful transition from *in vitro* to *in vivo* research.

### GBOs

Although 2-D and 3-D cell models and disease-relevant animal models have profoundly advanced GBM research, they must be improved in some aspects. Hence, newly developed 3-D organoids have gained substantial attention from GBM researchers^3^. In 2013, Lancaster et al.^13,14^ first formally proposed the concept of 3-D human brain organoids. The researchers used Matrigel to mimic the tissue around the developing brain and rotating bioreactors to help absorb nutrients and diffuse oxygen, thereby forming defined brain regions and enabling retrieval of a complex combination of brain cells and brain-like microstructures in a relatively short time.

Furthermore, Park et al.^15^ have developed a simple matrix-free vivo-like GBO model with patient-derived xenografted GBM cell lines in a small bioreactor. The authors have optimized the shear stress to reproducibly generate GBOs > 1 mm in diameter within 4–5 weeks. Scanning electron microscopy, immunohistochemistry, and single-cell transcriptomics have indicated that GBM cells can trans-differentiate into endothelial cells, pericytes, astrocytes, and self-established layered tissue structures, and even establish a heterogeneous tumor microenvironment (TME). GBOs exhibit high stemness and strong cell-cell interaction, and recapitulate GBM-TME characteristics *in vivo*, thus suggesting that uniformly sized GBOs could be produced and providing a reference for developing improved *in vitro* GBM models.

### Genetically manipulated cerebral organoids

To increase the success rate of organoid culture, researchers have explored improved methods for culturing organoids. A protocol has been developed for genetically manipulating brain organoids to efficiently induce GBOs; the model has been named neoplastic cerebral organoids (neCORs). The model can be genetically modified with the CRISPR/Cas9 technique, thus enabling the study of specific roles of essential genes associated with GBM pathogenesis^16^. Ogawa et al.^17^ have used CRISPR/Cas9 technology to achieve targeted integration of the hRASG12V-IRES-TdTomato construct at the TP53 genomic locus, thereby blocking TP53 protein activity in established neCORs. A distinctive feature of the neCOR model, as compared with other *in vitro* brain tumor models, is that tumors can be triggered by the introduction of genetic aberrations into a very small subset of cells in brain organoids. This model has the advantage of not only mimicking the onset of human tumors *in vivo* but also resulting in a complex structure that mimics human pathology and consists of tumors surrounded by normal tissues. The system is complementary to other models and clinical studies, thus aiding in elucidation of the molecular mechanisms of tumorigenesis, invasion, and progression of cancers. Moreover, this model enables high throughput validation of potential drugs and biological therapeutic agents, and supports exploratory drug discovery^18^.

Loong et al.^19^ have reported a proof-of-concept study for the personalized treatment of GBM with GBOs. The authors have presented a case of GBM in which a portion of the tumor tissue isolated after surgery was used to establish the GBO. The other portion of the tumor was used for targeted sequencing to quantify the expression of GBM-associated genes and develop drug targets. RNA sequencing analysis revealed mutations and loss of PTEN gene copies, thus suggesting activation of the common mTOR pathway in GBM. Next, the authors used patient-specific GBOs to screen a group of FDA-approved mTOR inhibitors for GBM treatment. The therapeutic effects achieved in GBOs were similar to those in patients with GBM. Furthermore, another study has generated iPSCs from patients with mutations in the c-Met proto-oncogene and found that iPSCs carrying this mutation spontaneously differentiate into dopaminergic neurons more rapidly than control iPSCs. By day 90, the gene expression profiles of c-Met mutated iPSCs showed increased expression of neuronal and GBM-associated genes. c-Met mutant iPSC-derived GBOs showed higher expression of astrocyte-specific GFAP and higher levels of phosphorylated c-Met and STAT3 than control samples. Drug testing indicated that TMZ had an better treatment effect on c-Met mutated neurons^20^. These findings have demonstrated the promising applications of *ex vivo* organoid models to achieve personalized therapy for patients with GBM. However, the high experimental costs remain a potential setback in the use of this model^21^.

### Patient-derived organoid (PDO) models

In 2016, Hubert et al.^22^ developed and optimized *in vitro* conditions to culture GBOs from patient-derived GBM cells, xenografted gliomas, and genetically engineered mouse gliomas. In 2020, Jacob et al.^23^ reported an even faster protocol for constructing GBOs. Compared with the 2 months in Hubert et al.^22^ and 20–30 days in Lancaster et al.^13^, this protocol can complete organoid culture in 1–2 weeks. Jacob et al. have established 70 organoid strains from a cohort of 53 patients with GBM. Transcriptomic profiling, exome sequencing, and single-cell transcriptomic analyses indicated that the GBOs preserve both intra- and intertumoral heterogeneity. These GBOs retain many key features of their corresponding parental tumors, displaying rapid and aggressive infiltration and maintaining key driver gene expression, such as Sox-2, Olig2, and EGFR. Furthermore, co-culturing of GBOs with chimeric antigen receptor-T (CAR-T) cells has revealed the potential of tumor organoids in immunotherapy screening^24^. Linkous and Fine^25^ have reported a standardized culture method, including sample preparation, culture conditions, feeding passage methods, embryoid body production, neural rosette knots, brain tissue development, brain organoid glioma tumor generation, and a series of other processes, in relation to GBO formation.

Liang et al.^26^ have innovatively used a biomimetic guidance system with light-curable hydrogels. They have reported significantly enriched osteoclastic differentiation and lysosomal pathways, but not cytokine-cytokine receptor interaction pathways, in the hydrogel-containing GBO group compared with the control group. Moreover, RNA sequencing evaluation of the expression profiles of essential genes associated with the biological characteristics of GBOs at the transcriptomic level has indicated that GBOs cultured with hydrogel retain an expression profile of crucial neurodevelopmental markers, driver mutations, and selective splicing patterns of parental tumors. Oudin et al.^27^ have obtained small tumor fragments (approximately 0.5 mm × 0.5 mm) invisible to the naked eye by collecting brain tumor tissue from patients with GBM and mincing the tumor tissue with a sterile scalpel at 20–22 °C in a secondary biosafety cabinet. After culturing, effectively derived organoids were obtained. The organoids were subsequently implanted intracortically in immunodeficient mice to induce tumor growth *in vivo*, thus resulting in successful establishment of a PDO model of GBM. PDO models derived from primary and metastatic brain tumors recapitulate clinical patients’ responses, and can be used to identify adjuvant therapeutics to achieve favorable outcomes in patients with relapse and to determine efficient treatment options, thereby improving patient survival^28^. Glioma PDOs can maintain tumor stages over long time periods, and provide clinically relevant patient models that preserve the histopathological, genetic, epigenetic, and transcriptomic features of the parental tumors. Patient-derived orthotopic xenograft (PDOX)-derived standardized glioma organoids can be adapted for high-throughput drug screening and validation, and used instead of mouse models^29^. Zhang et al.^30^ have systematically simulated the histological characteristics, responses to chemotherapeutic agents, and clinical progress in corresponding parental tumors through an integrated system, on the basis of a parallel model of 3-D *ex vivo* brain organoids and *in vivo* xenografted tumors generated from GBM patient-derived cell lines. This model may advance understanding of glioma biology and the prediction of patient responses to chemotherapy drugs. Therefore, PDOs are a promising as a new strategy for the personalized treatment of gliomas.

### Cerebral organoid-brain cell interaction

In 2018, da Silva et al.^31^ demonstrated that glioma stem cell (GSC)-derived spheroids can attach to and invade immature brain organoids. That work provided the first evidence that patient-derived cells interacting with brain organoids can be used as a GBM model. Linkous et al.^10,32^ have established the cerebral organoid-brain cell interaction (GLICO) model and co-cultured GSCs or human embryonic stem cell (hESC)-derived GBOs. The GLICO model offers additional advantages over both the GBOs and neoCOR models. Because GLICO is created with patient-derived GBM cells and brain organoids (possibly from the same patient), it provides a unique ability to study tumor-brain interactions. In contrast, the GLICO model has similar weaknesses to those of other GBO models, because of the lack of vascularization and immune cells. Krieger et al.^33^ have proposed an experimental model combining organoid and single-cell transcriptomic analyses to study the cellular interactions of aggressive GBM cells in human brains. In this model, iPSC-derived human brain organoids are used as a 3-D scaffold for patient-derived GBM cell invasion. The model can be established in fewer than 4 weeks and used to quantify tumor tubule development, on the basis of tissue clearance, confocal microscopy, and semi-automated quantitative analysis. Moreover, single-cell RNA sequencing analyses of samples before and after co-culturing of GBM cells and normal brain cells have revealed a set of differentially expressed transcripts, which may serve as potential therapeutic targets for GBM. Fawal et al.^34^ have observed DHFR overexpression in several human brain tumors as well as in human brain tumor-initiating cells (BTICs). Studies have shown that either direct inhibition of DHFR with methotrexate or transcriptional inhibition of DHFR with synthetic ligand-activated EphB decreases the self-renewal ability of 4 human BTIC lines *in vitro* and the GLICO model. These results suggest that driving BTIC differentiation by inhibiting DHFR may serve as a new treatment for highly refractory aggressive tumors. The GLICO model uniquely enables study of tumor-brain interactions, thus allowing for analysis of tumor development by mimicking the brain tissue microstructure^35^.

### 3-D GBM model development *via* bioprinting

Initial GBM research focused on the development of xenograft assays. Since then, technological advancements have allowed tumor cells to be efficiently dissociated into single-cell suspensions, to establish neurosphere culture and facilitate selective expansion. Moreover, genes involved in GBM tumorigenesis and progression have been identified and characterized, thereby leading to the generation of GBM mouse models. Recent advances include mini-brains with GBOs or 3-D bioprinting^36^. The 3-D bioprinting technology enables the fabrication of user-defined 3-D objects guided by a computer-aided design model, and high resolution, repeatability, flexibility, and customizability make this model widely applicable in biomedical research^37^.

**Figure 1:**
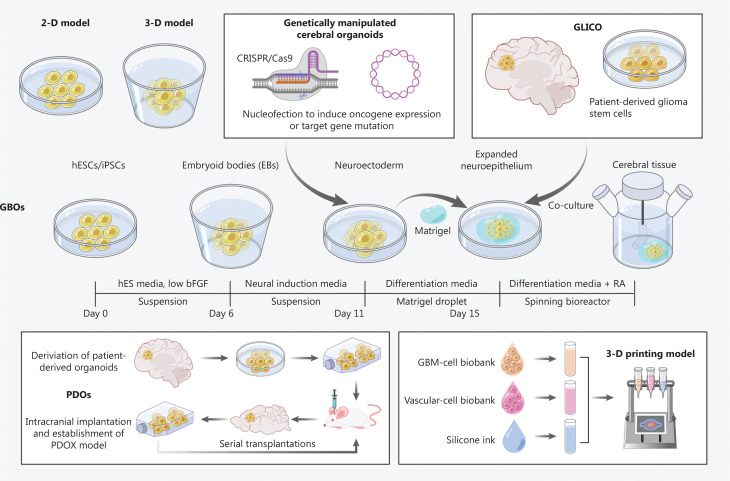
Diagram of the construction patterns of different GBOs. The *in vitro* model of GBM was developed from the original 2-D and 3-D models and translated to the GBO model, and the genetically manipulated cerebral organoids, GLICO, PDO, and 3-D printed models were derived from the GBO model.

In 2019, Yi et al.^38^ created a GBM chip bioprinted with reconstituted GBM tumors consisting of patient-derived tumor cells, vascular endothelial cells, and decellularized ECM. The model sustains a radial oxygen gradient and recapitulates the structural, biochemical, and biophysical properties of native tumors. Patient-specific resistance with chemoradiotherapy and TMZ therapy has been observed in the bioprinted model. This patient-specific tumor-on-a-chip model might aid in determining effective treatments for patients with GBM resistant to the standard first-line treatment. Hermida et al.^39^ have used another bioprinting method to generate a multilineage GBM model and have shown its advantages in testing drugs with fluorescently tagged protein kinase reporter genes for cell signaling analysis. Other authors have used biomaterials such as hyaluronic acid (HA)-based hydrogels and synthetic polymers such as polyethylene glycol for the same purpose^40^. Recently, other groups have further improved the GBM model of bioprinting. Maloney et al.^41^ have reported a novel immersion bioprinting method to facilitate drug screening with bioprinted GBM organoids. Tang et al.^42^ have combined multiple cell types to develop a “four cultures” system including bioprinted macrophages to mimic immune interactions. However, the system still requires an accurate 3-D spatial organization, which can be generated only with self-assembled 3-D organoid cultures, and also requires advanced technologies and expertise rarely available in most standard biological laboratories. In another study, van Pel et al.^43^ have combined 3-D printing technology with 3-D brain organoids to develop a system mimicking the invasion of human glioma cells into mouse neural progenitor cell-derived spheroids, thereby enabling real-time and fixed sample tracking of human tumor cell invasion with cell tracking dyes and 3-D laser scanning confocal microscopy, and simulating differences that might be observed in the brains of patients with GBM. Because conventional models cannot predict therapeutic effects on GBM, *in vitro* 3-D GBM models reconstructed by 3-D bioprinting with biomaterials and patient-derived cells may be applied recapitulate the basic physiological and pathological features of GBM. These 3-D bioprinted GBM models enable species-matched, high-throughput, and reproducible investigation of cell-ECM interactions, drug screening, and optimization of tumor-specific drug delivery^44^. The construction patterns of various GBOs are shown in **Figure 1**.

## Application of organoids in GBM research

### Biological function studies

Brain organoids have been found to have good application value in studying the aggressive growth, inflammation, metabolic level, and niche microenvironment of GBM. Zhang et al.^45^ have demonstrated that dendritic polyglycerol sulfate might be an effective therapeutic agent that decreases inflammatory markers and GBM aggressiveness by modulating microglial activity in a 3-D reconstructed GBO model. Shakya et al.^46^ have performed RNA sequencing analysis of lipid-associated gene expression in separate microenvironments in the GBO model. They have found significant differences in the expression of lipid processing genes and total lipid content across different cell populations in the same patient; lipid enrichment in the hypoxic organoid core; and necrotic features of tumors in Patients with primary GBM. Fatty acid desaturase (FAD) is upregulated in GSC, and FADS1 and FADS2 are essential for the survival and maintenance of GBM GSCs, thus indicating that the changes in lipid levels might reflect not only the TME but also the cellular state. The GBO model recapitulates the clinically distinct lipid metabolism phenotypes found in most patients with GBM. Brain organoids have promising applications in simulating the aggressive growth characteristics of tumors. Liu et al.^47^ have used human brain organoids as 3-D tissue substrates to knock down the oncogenic gene lncGRS-1 (screened with CRISPRi technology) expression in GBM, thereby selectively decreasing tumor growth and sensitizing glioma cells to radiation therapy. Moreover, another study has used the CRISPR-Cas9 technique to transform EGFRvIII-mutated and wild-type organoids into H9-hESCs. The EGFRvIII-mutated organoids are larger, retain more astrocytes, and have higher Ki67 expression than wild-type organoids, thus suggesting that EGFRvIII mutation enhances cell proliferation. EGFRvIII- organoids show significantly increased apoptotic cell death after TMZ treatment, thus indicating that this model can also be applied to evaluate anti-GBM drugs^48^. In addition, GBOs have essential applications in studying glioma cell niche microenvironments. Quiescent cells have been associated with glioma recurrence and treatment resistance, but difficulties in their direct visual identification and a lack of targeted therapy have hindered their mechanistic investigation. By using genetic tools to visualize and ablate quiescent cells in mouse brain tumors and human glioma organoids, localization of quiescent cells at the core and edges of tumors has been revealed, thus suggesting that these cells might be involved in tumor cell infiltration^49^. Another study has used a 3-D GBM organoid approach to explore self-renewal capacity, high therapeutic resistance, and mesenchymal gene signatures in quiescent cell populations. Bioinformatics analysis and functional assays of GBOs have identified hypoxia and TGF-β signaling as potential niche factors promoting GBM quiescence, mesenchymal transfer, and interaction with the niche microenvironment^50^.

### Research on drugs for GBM treatment

Chemoradiotherapy has positive effects on the prognosis of patients with GBM^51^. Organoids can be extracted from patients’ tumor tissues, thus preserving the histological properties of the parental tumors and consequently providing a valuable *in vitro* model for drug screening^52^. Many research teams have conducted proof-of-concept experiments demonstrating the feasibility of this strategy in drug discovery. Some researchers have used patient-derived GBM organoids to validate antitumor compounds with high-throughput screening *in vitro*^53,54^. Establishment of a biobank of relevant tumor organoids allows researchers to perform gene sequencing analysis to capture disease heterogeneity and to use high-throughput drug screening techniques to identify potential druggable targets^55^.

Recently, multiple studies have reported the successful application of GBO models in therapeutic drug development. Wei et al.^56^ have used Gamitrinib, an HSP90 inhibitor, to regulate mitochondrial localization, thus inhibiting cell proliferation and inducing apoptosis in 3 different types of organoid models, including GBOs. The novel BET inhibitor UM-002 decreases GBM cell proliferation and invasion in human brain organoid models^57^. Bayat et al.^58^, by co-culturing GBOs with human umbilical vein endothelial cells in fibrin gel, have found that atorvastatin inhibits angiogenesis and downregulates the expression of VEGF, CD31, and Bcl-2. Thus, atorvastatin may be a promising drug for treating GBM. TMZ is currently the most commonly used and effective drug for GBM in clinical practice, but drug resistance in patients is almost inevitable. Yi et al.^38^ have demonstrated that bioprinted GBM organoids can be used to determine the therapy sensitivity in PDOs to overcome TMZ resistance. A major mechanism of TMZ resistance is abnormalities in the DNA damage response. The authors have defined 2 different primary transcription profiles with microarrays to detect GBM samples, identified abnormal DNA damage responses in GBM, and subsequently demonstrated that DNA repair inhibitors and radiotherapy are more effective against cells that respond abnormally to DNA damage on GBOs. Reed et al.^59^ have used a GBO model to assess the susceptibility of patients with GBM to Li Fraumeni syndrome (LFS). They have found that patients with GBM and LFS have significantly elevated STAT1 and STAT2 expression, according to comparative transcriptomic analysis. GBOs from patients with LFS express higher levels of STAT1 and STAT2 than other GBO models and are more sensitive to Ruxolitinib treatment. Yi et al.^60^ have revealed the role of the RNA-binding protein Msi1 in treating GBM by using a GBO model. In GBM and other tumor types, Msi1 expression levels are generally elevated and are associated with poor clinical outcomes. In addition, Msi1 is associated with chemo-radio resistance. The authors have found that luteolin, an inhibitor that suppresses the binding of Msi1 to oncogens and reduces their stability and translation, successfully decreases the proliferation of patient-derived glioma initiation cells and GBOs. Lenin et al.^61^ have developed a novel drug screening scheme using 2-D cultures and patient-derived 3-D GBOs. A total of 65 drugs/inhibitors have been screened, and Vismodegib, Disulfiram, Parthenolide, Omipalisib, and Costunolide have been found to have the most significant effects on GSC viability. Furthermore, the TERT inhibitor Costunolide has been found to effectively decrease cell viability in primary tumors and tumors pretreated with chemo- and radiotherapies. To study the activity of Monensin (MON) analogs in the TME, Urbaniak et al.^62^ have developed human brain organoids with iPSCs. MON analog 1 decreased U87MG tumor size in GBOs after 4 days of treatment and induced a significant decrease in PARP expression, thus indicating the potential anticancer effects of MON analogs on GBM. Darrigues et al.^12^ have evaluated the efficacy of drugs and small molecule inhibitors in preventing glioma invasion by using PDOs for screening; 2 tubulin inhibitor compounds (ST-145B and PNR-4-44) and 1 COX-2 inhibitor (PNR-5-82) were found to have favorable inhibitory effects, and tubulin inhibitor compounds had the best efficacy in U-251 MG cells. Although the efficacy of compounds in *ex vivo* patient organoids is highly correlated with intra and intratumor heterogeneity, a combination of spheroid and GBO models can be used to determine the invasion inhibition efficacy of many compounds. The GBO model can be used to study patient-specific responses to immunotherapy *via* co-culture with CAR-T cells^63^. Schnalzger et al.^64^ have used EGFRvIII-expressing CAR-NK-92-derived organoids to analyze tumor antigen-specific cytotoxicity and evaluate the relationship between the efficacy of CAR-T therapy and tumor specificity. CAR-modified NK-92 cells directly target epithelial cell adhesion molecules, thus enabling effective targeting of multiple organoids simultaneously. Furthermore, GBOs can be used to study GBM-targeted drug delivery across the blood-brain barrier^65^. These results suggest the importance of rapid, functional testing of GBOs in achieving personalized drug therapy.

### GSC research

GSCs have been identified as a treatment-resistant tumor component promoting tumor growth and progression. GSCs are believed to be responsible for cancer recurrence after treatment. Despite the critical roles of GSCs, suitable preclinical models for studying the genetic and epigenetic markers driving their malignant behaviors within the TME remain lacking^53^. The introduction of 3-D tumor platforms, such as organoids and 3-D bioprinting, represent the pathophysiological interactions between glioma GSCs and TME^66^. Glioma organoid technology also enables investigation of GSC invasion in human brain tissues, thus mimicking a suitable brain-like microenvironment^67^. Benitez et al.^68^ have performed high-throughput drug screening on GSCs in the GBO model and have found that GSCs are highly sensitive to proteasome inhibitors, owing to potential dependence on the increased protein synthesis rate and loss of autophagy-associated PTEN and PI3K/mTOR pathway activation. Proteasome inhibition specifically increases the expression of cell death markers in 3-D GBOs, inhibits tumor growth, and increases survival in mice transplanted with GSCs *in situ*. Using organoid-based glioma invasion assays and mouse brain xenografts, Goranci-Buzhala et al.^69^ have shown that patient-derived GSCs recruit high levels of proteins, thereby ensuring temporal ciliary breakdown and inhibiting ciliogenesis. Depletion of cillolytic complex components is sufficient to induce production of cilia in the GSC subpopulation by relocating platelet-derived growth factor receptor-α (PDGFR-α) to newly generated cilia, whereas restoration of ciliary production enables GSCs to shift from a self-renewal to a differentiated stage. Ciliary production-induced differentiation has been found to prevent GSC infiltration into the brain. Innes et al.^70^ have used organoids to reveal that different microenvironments produced by the transformation of GSC clonal populations from adherent culture to organoids have different clonal phenotypes and are highly plastic. Zhu et al.^71^ have used brain organoids to study the oncolytic effect of the Zika virus and have observed that this virus preferentially infects and kills GSCs rather than neural progenitor cells. In addition, GBOs contribute to the development of immunotherapeutic targets and drug screening for GSCs. Knockdown of the m6A-modified pseudogene HSPA7 has been found to increase the efficiency of anti-PD1 therapy in the GBO model^72^. The GBO model has also played a critical role in therapeutic research on biological peptides. New treatments applying bioactive peptides may contribute to the multiplex targeting of GBM, particularly GSCs. Biopeptide-based therapeutics must use advanced GBM *in vitro* models, such as organoids, to explore combination approaches for clinically including bioactive peptides to standard cancer treatments^73^. Brain organoids have also been reported to be applied to the latest electric field therapy, and have shown good auxiliary value in evaluation of the efficacy and safety of electric field therapy^74^. The research applications of GBOs are detailed in **Table 2**.

**Table 2:** Applications of organoids in GBM research

Application	Targets	Mechanisms	References
Biological function study	Inflammation	Dendritic polyglycerol sulfate limits GBM invasiveness by modulating microglial activation in GBOs.	^45^
	Lipid metabolism	GSCs have lower lipid droplet accumulation than non-GSCs in GBO models and xenograft tumors.	^46^
	Invasion	lncGRS antisense nucleotides decrease aggressive growth of tumors in GBOs.	^47^
	Proliferation	EGFRvIII mutation-induced astrogenesis and massive cell proliferation in human GBOs are observed.	^48^
	Microenvironment	Quiescent cells are partially responsible for tumor cell infiltration and invasion in GBOs.	^49,50^
Drug treatment	Gamitrinib	Gamitrinib inhibits cell proliferation, and induces cell apoptosis and death, in 17 primary glioma cell lines, 6 TMZ-resistant glioma cell lines, 4 neurospheres, and 3 PDOs.	^56^
	UM-002	The novel BET inhibitor UM-002 decreases glioblastoma cell proliferation and invasion in GBOs.	^57^
	Atorvastatin	Atorvastatin has potent anti-angiogenic and apoptosis inducing effects against glioma spheroids.	^58^
	Temozolomide	Bioprinted GBOs reproduce clinically observed patient-specific resistance to treatment with concurrent chemoradiation and temozolomide.	^38^
	Ruxolitinib	Patients with GBM with than without LPS expression have higher levels of STAT1 and STAT2, and are more sensitive to ruxolitinib therapy.	^59^
	Luteolin	Luteolin decreases the proliferation of patient-derived glioma initiating cells and tumor organoids, but does not affect normal astrocytes.	^60^
	Costunolide	The TERT inhibitor costunolide effectively decreases cell viability in both primary GBO models and GBO models pre-treated with chemotherapy and radiotherapy.	^61^
	MON	MON decreases U87MG tumor size in GBOs and significantly decreases PARP expression.	^62^
GSC research	PTEN	GSCs are highly sensitive to proteasome inhibition, owing to an increased protein synthesis rate and loss of autophagy, associated with PTEN loss and activation of the PI3K/mTOR pathway.	^68^
	Ciliogenesis	Ciliogenesis-induced differentiation prevents the infiltration of GSCs in GBOs.	^69^
	Phenotype	Different microenvironments produced by transfer of GSC clonal populations from adherent culture to organoid conditions have different clonal phenotypes and are highly plasticized.	^70^
	ZIKV	ZIKV selectively eliminates GSCs from species-matched human mature cerebral organoids and GBM surgical specimens, but this effect is reversed by integrin αvβ5 inhibition.	^71^
	ICB therapy	HSPA7 promotes macrophage infiltration and SPP1 expression *via* upregulating YAP1 and LOX expression in GSCs. Knockdown of HSPA7 increases the efficiency of anti-PD1 therapy in the GBO model.	^72^

## Deficiencies in GBOs and methods for improvement

GBOs are promising high-fidelity models for disease modeling, drug development, living biobank building, treatment response simulation, and exploration of personalized treatment. However, GBO models face several challenges, including insufficient immune response, difficulty in innervation, lack of a vascular system, controversy regarding their reliability, control of the biophysical and extraphysical organoid environments, and modeling of tissue-tissue linkages. These difficulties may hinder research on organoids^54^. Importantly, a major limitation of brain organoids is the inclusion of multiple ventricular zone-like regions of varying sizes, which can hinder the reproducibility and quantification of cellular structures^75,76^. Moreover, because of the lack of the ordinarily present embryonic environment (particularly a body axis), although organoids can develop into discrete brain regions, they do not form the same patterns that occur under normal physiological conditions^77^. Another drawback is the lack of an immune response, which is particularly important for GBM modeling^78^. The diffusion of nutrients inside brain organoids to cells is also a critical issue; when organoids grow beyond a specific size without forming a vascular network, the center of the organoid may undergo necrotic cell death^79^, because cells more than 200–400 μm from the cell surface cannot obtain sufficient oxygen and nutrients through diffusion alone^80^.

In recent years, substantial efforts have been made to establish PDOs that preserve parental tumor heterogeneity, relative 3-D spatial organization, and fundamental interactions with the ECM^81–84^. The vascularization of brain tissue is a key factor in developing effective brain organoids^85^. Recently, several methods have been developed to induce brain organoid angiogenesis *in vitro*. One approach includes genetically modified human iPSCs expressing ETV2 in the cell population to induce brain organoid formation. Over time, stem cell differentiation and maturation can create vascular-like structures in organoids^56^. Another method has been introduced during EB formation^85^. Furthermore, human endothelial cells can be embedded in Matrigel and added to early organoids. This approach allows human endothelial cells to self-assemble into capillaries on the periphery of organoids and efficiently invade vascular networks over time^80^. In all cases, substantial vascular network formation is observed on the periphery of the organoids, whereas less of a vascular network is seen toward the center. Transcriptomic analysis has confirmed enhanced angiogenesis and upregulation of angiogenesis-associated genes^85^. Finally, introduction of immune cells (microglia) can increase the ability of brain organoids to recapitulate brain tissue responses to GBM invasion^86^. With the continued development of organoid technology, newer organoid culture methods, such as 3-D printing, have been able to better simulate tumor invasion patterns and drug resistance by introducing monocytes, astrocytes, and GSCs^38,40^, and have enabled progress in preclinical drug testing^41^.

## Future perspectives

Basic research on tumors is important for the prevention and treatment of diseases^87^. Organoids provide a valuable platform for investigating organ development and mimicking pathological processes. Compared with the traditional models of 2-D cell lines and PDOX models, organoids have unique advantages in drug screening and precision medicine^88^. Because organoids preserve the heterogeneity among patients, researchers have recognized their importance in clinical screening. Organoids and the resulting PDOX models recapitulate patients’ tumor-specific histological, genetic, epigenetic, and transcriptomic features^29,89^. Organoids and PDOX can be directly applied in various functional assays, such as biomarker discovery^90^, tumor heterogeneity assessment^91^, cell type-dependent TME models^92^, and immune interactions^42^. Moreover, brain organoids induced by GBM pathology, particularly organoids generated by patient-derived cells, have been used to test the therapeutic efficacy of various treatments, such as radiotherapy^22^ and chemotherapy^93^. Organoid models can be exploited for functional analysis of anticancer drugs^94^ for preclinical screening and identification targeting specific glioma subtypes, thus providing an effective platform for drug discovery^95^. Future research will focus on developing protocols to rapidly generate patient-specific organoids so that they can be used to test the efficacy of existing clinical treatments, thereby providing a basis for patient-specific drug selection. Organoid-based drug screening may serve as a promising pathway for the precision treatment of patients with GBM^96^.

Despite the many limitations of brain organoids, GBOs is the most appropriate *in vitro* model to reproduce patient heterogeneity. Additionally, emerging technologies such as 4-D real-time imaging, microfluidics, organ-on-a-chip technology, and single-cell sequencing are also important methods of improving organoid models. Overall, organoid technology has opened new avenues in GBM therapy, GBO has gradually become an ideal preclinical model in GBM research. Tumor organoid models have a high success rate *in vitro* culture, owing to their short formation time, low cost, stable passaging, ability to recapitulate the *in vivo* tumor characteristics and heterogeneity of the primary tumor, easy genetic screening, and ability to be revived after cryopreservation. These characteristics have enabled the establishment of various tumor organoid sample information databases, thereby providing sufficient data support for experimental studies. With improvements in *in vitro* culture technology and the construction of various tissue organoids, the strong momentum for development has made organoids a hotspot in biomedicine research. Organoid models are expected to substantially enable the exploration of disease mechanisms, personalized treatment, drug development, and regenerative medicine, and to play an increasingly important role in tumor research advancement, continuing technological innovation, and further development of organoid biobanks and chips.
